# Implementation barriers and remedial strategies for community-based health insurance in Bangladesh: insights from national stakeholders

**DOI:** 10.1186/s12913-022-08561-7

**Published:** 2022-09-24

**Authors:** Nurnabi Sheikh, Eunice Twumwaa Tagoe, Raisul Akram, Nausad Ali, Susan Howick, Alec Morton

**Affiliations:** 1 Department of Management Science, Strathclyde Business School, University of Strathclyde, Glasgow, UK; 2 Institute of Health and Wellbeing, Health Economics and Health Technology Assessment, University of Glasgow, Glasgow, UK; 3 Population Studies Division, Bangladesh Institute of Development Studies (BIDS), Dhaka, Bangladesh

**Keywords:** Community-based health insurance, Implementation barriers, National health protection scheme, Universal health coverage, Bangladesh

## Abstract

**Background:**

Community-based health insurance (CBHI) is a part of the health system in Bangladesh, and overcoming the obstacles of CBHI is a significant policy concern that has received little attention. The purpose of this study is to analyze the implementation barriers of voluntary CBHI schemes in Bangladesh and the strategies to overcome these barriers from the perspective of national stakeholders.

**Methods:**

This study is exploratory qualitative research, specifically case study design, using key informant interviews to investigate the barriers of CBHI that are faced during the implementation. Using a topic guide, we conducted thirteen semi-structured in-depth interviews with key stakeholders directly involved in the CBHI implementation process. The data were analyzed using the Framework analysis method.

**Results:**

The implementation of CBHI schemes in Bangladesh is being constrained by several issues, including inadequate population coverage, adverse selection and moral hazard, lack of knowledge about health insurance principles, a lack of external assistance, and insufficient medical supplies. Door-to-door visits by local community-health workers, as well as regular promotional and educational campaigns involving community influencers, were suggested by stakeholders as ways to educate and encourage people to join the schemes. Stakeholders emphasized the necessity of external assistance and the design of a comprehensive benefits package to attract more people. They also recommended adopting a public–private partnership with a belief that collaboration among the government, microfinance institutions, and cooperative societies will enhance trust and population coverage in Bangladesh.

**Conclusions:**

Our research concludes that systematically addressing implementation barriers by including key stakeholders would be a significant reform to the CBHI model, and could serve as a foundation for the planned national health protection scheme for Bangladesh leading to universal health coverage.

**Supplementary Information:**

The online version contains supplementary material available at 10.1186/s12913-022-08561-7.

## Background

Developing nations are increasingly interested in exploring alternative healthcare financing systems to improve health coverage, accessibility, and financial protection to achieve universal health coverage (UHC). In the early 1980s, many developing countries introduced alternative cost recovery public health financing via a user fee system [1]. However, user fees limit healthcare-seeking ability, particularly for those living in the countryside. Many commentators, therefore have been calling for an effective prepaid health financing mechanism to eliminate financial barriers associated with healthcare access [2]. Health insurance has been proven effective at increasing access to healthcare and protecting individuals and families from catastrophic health expenditures. The underlying modality of health insurance allows beneficiaries to pay for healthcare in advance to avoid out-of-pocket expenditure at the point of care. Social health insurance (SHI) is a form of health insurance usually provided by a central government and sometimes by non-government organizations (NGOs) at a community level [3]. SHI in low-and-middle-income countries (LMICs) which rely heavily on taxes may face implementation barriers due to inadequate tax collection, low institutional capacity to collect taxes, and a large informal sector workforce. Another form of health insurance is community-based health insurance (CBHI) which proliferated throughout developing countries, especially in Africa in the early 1990s [4]. CBHI schemes are also referred to as mutual health insurance schemes, local health insurance, micro-health insurance, and medical aid societies or medical aid schemes [5]. These insurance schemes are typically managed by local NGOs, hospitals, civil society organizations, or organized cooperative societies with community participation in their management [6]. The primary objective of CBHI schemes is to mobilize local resources to provide quality healthcare services and increase healthcare accessibility in deprived areas [7]. Ghana, Rwanda, Japan, and China started with CBHI and then reformed their health financing system by integrating CBHI schemes into national health insurance; however, their journey to form national health insurance from the CBHI schemes was not straightforward [5, 8–10]. Mali, Senegal, and India are exploring a similar CBHI scaling-up strategy [10].

Historically, Bangladesh had a tax-based healthcare system; however, the dearth of government capacity and financial protection schemes has resulted in a severe burden of out-of-pocket health expenditures [11]. In Bangladesh, CBHI exists mainly as a form of micro-health insurance initiated by microfinance institutes, NGOs, and hospital/healthcare providers. CBHI in Bangladesh is mainly affiliated with microfinance institutes, where microfinance operators launched health insurance for their borrowers to ensure their well-being. In the late 1990s and early 2000s, microfinance institutions began offering health insurance to their borrowers [12]. The pioneer organizations for CBHI are Gonoshasthaya Kendra, Ad-din, Grameen Kalyan, Bangladesh Rural Advancement Committee (BRAC), Diabetic Association of Bangladesh, Sajida Foundation, Dhaka Community Hospital, Shakti, Nari Uddug Kendra, Dushtha Shasthya Kendra, Integrated Development Foundation, Society for Social Services and International Centre for Diarrhoeal Disease Research Bangladesh [12, 13]. We might broadly categorize CBHI based on the types of insurance providers: (i) provider-based model, in which private health facilities commence health insurance and offer healthcare from their health facilities; (ii) microfinance-based model, where microfinance organizations manage insurance programs for their borrowers; and (iii) non-microfinance-based model, where NGOs launch health insurance for the organized community or specified geographic areas without any link with microfinance [13]. In a provider-based model, private health facilities act as both insurers and providers, similarly, most microfinance institutions also use their health facilities to provide healthcare [13, 14]. Non-microfinance NGOs most often buy health care from private healthcare providers, but some also buy insurance packages from insurance companies [13]. The insurance company then provides health care from its contracted health facilities [15]. Enrolment is generally compulsory in a microfinance-based model for their borrowers, whilst provider-based and non-microfinance-based models allow voluntary enrollment. Most CBHI schemes treat the family/household as an enrollment unit. CBHI offers discounts for poor and vulnerable communities and has a greater emphasis on maternal and child healthcare. However, most of these schemes are highly dependent on external funding and/or provide cross-subsidies to recover fund gaps although some of these schemes are evidence to cover a certain amount of operational cost [12]. They also offered discounts on medicine and pathological tests however, most of them do not have referral systems. CBHI has been shown in several studies to enhance healthcare utilization among the rural poor and lessen out-of-pocket healthcare spending in Bangladesh [16–18].

Although CBHI schemes offer financial protection against healthcare costs to a group of people, the literature indicates that such a financing strategy is not much effective in the advancement of achieving UHC goals [7]. The key grounds for this argument are adverse selection, moral hazard and heavy reliance on external subsidy challenge scheme implementation [4, 7]. Major barriers of CBHI relate to conventional health insurance, including a small risk pooling, limited technical and organizational knowledge, low service coverage capacity, poor quality of care, and inadequate service providers [4].

Although there is substantial literature on CBHI in Africa and Asia, there are few studies in Bangladesh [14]. Most of the Bangladeshi literature focuses on the impact of CBHI on healthcare access and out-of-pocket spending and investigates the factors that influence enrollment and renewal [19–21]. Hence, this study aims to explore the implementation barriers of CBHI in Bangladesh, and strategies to overcome these barriers. Bangladesh is behind other countries in the journey to UHC and the adoption of CBHI as a mechanism of financing for healthcare. The Government of Bangladesh plans to reform healthcare financing, and CBHI is an integral part of the adopted healthcare financing strategy 2012–2032 [11]. To secure healthcare for the massive informal sector, the government strategy relies heavily on CBHI. In this context, it is crucial to understand CBHI's implementation barriers since this will benefit policymakers in their efforts to implement a healthcare financing strategy, particularly targeting the informal sector.

## Methods

### Study design

This case study was conducted in Bangladesh using in-depth interviews to investigate (i) barriers to CBHI implementation and (ii) strategies to overcome these barriers from the perspective of researchers, policymakers, and individuals directly involved in the operation of CBHI. We employed a case study design as the approach offers an opportunity to perform a thorough examination of the complex phenomena—CBHI—occurring in a particular environment, in Bangladesh [22].

### Participant selection and recruitment

We compiled a list of seven CBHI schemes with voluntary membership from the published literature. Only three of these schemes were operational during the data collection period, but we also wanted to look at the lessons learned from significant CBHI efforts that are no longer active. Fifteen key informants were purposively identified based on their experience and involvement in the seven CBHI schemes. Participants were selected based on who could provide the best information, using a method referred to as judgmental sampling or expert sampling. Interviews were conducted with the managers, field supervisors, and directors of the CBHI schemes due to their knowledge of the barriers to implementation. Email invitations that included the study information and consent form were sent to the fifteen potential interviewees. Nine people consented to participate in the study. These participants were from the Gonoshasthaya Kendra, International Centre for Diarrhoeal Disease Research, Bangladesh, BRAC, Grameen Kalyan, World Health Organization, World Bank and the University of Dhaka. Seven of the participants were actively involved in the implementation of CBHI schemes, while the remaining two were health financing policy experts and researchers. In addition to these nine participants, four officials from the Ministry of Health and Family Welfare were also interviewed to gain policymakers' views on CBHI schemes. There is widespread controversy regarding the ideal sample size for qualitative research, but most experts agree that data saturation is the most vital factor to consider in determining sample size for qualitative research [23–27]. In our study, we interviewed two groups of individuals. We covered implementation experiences from five of the seven recognized CBHI schemes in Bangladesh and ceased reaching out to additional participants once we assumed that no new information was being provided by our participants. We also interviewed two researchers to justify data saturation, i.e., to determine whether we missed any information on implementation barriers in addition to the information provided by our seven participants, but they did not provide any further insights. Then, we interviewed policymakers for our research to obtain their perspectives on CBHI. Similarly, once we understand that our interviewees have offered no new information, we stop recruiting new policymakers.

### Data collection

We conducted thirteen semi-structured personal interviews (including four government officials), using a topic guide, which was developed from a prior literature review conducted for this study. All study authors reviewed the guide for completeness and appropriateness of topics. Our participants were the CBHI implementers and researchers/policymakers. Therefore, to get maximum benefit the interview guide was piloted with a healthcare financing researcher who has experience in CBHI implementation. Interviews were conducted between March and June 2021. Seven interviews were conducted through online-based one-to-one interactive sessions and six were face-to-face with strict adherence to social distancing and COVID-19 protocols. Face-to-face interviews took place at the participants' workplace, either in an enclosed meeting room or in the participants' room to avoid interruptions during the interviews. Interviews commenced with the interviewer explaining the study's objective, and issues to be discussed and seeking verbal consent to audio record the conversation. The interviewer has prior experience in conducting in-depth interviews in Bangladesh. Participants were asked to describe their role and expertise and provide an overview of their schemes, including premiums, benefits packages, and enrollment procedures. The interviewer used an interview topic guide to know the barriers to CBHI implementation and probes to elicit more detailed information from the participants. The duration of the interviews ranged from 30 to 50 min. The interviews were carried out in Bengali and then translated into English.

It should be noted that the interviewer had a prior working relationship with the interviewees. This relationship may have influenced how questions were asked, reactions to interviewee responses and data analysis, making it challenging to be entirely objective, to listen solely from a researcher’s perspective and to take an outsider's perspective. The interviewer, however, was conscious of the need to maintain objectivity throughout the interview by reserving his own opinions and responses.

### Data analysis

Data were analyzed using the Framework Analysis method, which includes seven steps [28]. First, we translated the audio interviews from the exact form of speech in Bengali to English. Secondly, at the familiarization stage, the researcher read interview transcriptions repeatedly to gain more insights. Thirdly, in the coding phase, two researchers (NS and ETT) independently coded the same two randomly selected interviews based on the initial deductive codes identified from the literature review [20, 21, 29–32]. This led to the development of inductive codes by revising deductive codes from the two coded interview transcripts. In the fourth step, we developed a comprehensive working codebook framework based on the revised codes (Table 1) and applied this to the remaining eleven transcripts at the indexing (fifth) stage. Then, we developed a framework matrix using a spreadsheet with rows for interviewees and columns for codes and applied this to all interview transcripts to summarize data at the charting (sixth) stage, where each cell contained summarized data. The framework matrix was circulated to all researchers for review. It was subsequently updated in response to their suggestions. All researchers eventually reached a consensus on the final version of the framework matrix. The last step involved interpreting the matrix based on the study objectives.

**Table 1:** Codes and description

Codes	Description
Inadequate population coverage	Barriers as a result of low enrolment and low renewal rates, or the voluntary nature of scheme
High claim rate	Significant rate of healthcare consumption due to adverse selection and moral hazard
High startup and administrative cost	Regular advertising campaigns, transportation, claim processing, staff salaries, infrastructure, and so on all incur costs
Scheme design	Barriers due to benefit package design, premium amount, co-payment rate, etc
Competitive healthcare market	In the catchment areas, the influence of informal providers such as village practitioners, traditional healers, and drug sellers, as well as professional providers
Inadequate knowledge on health insurance principle and CBHI	Community's perception on health insurance modalities
Quality of care and trust	Barriers related to inadequate health care supplies e.g., healthcare providers and drugs availability, quality of drugs, healthcare providers behaviors, etc. and barriers to trust on management due to less community or government engagement or due to earlier bad experiences
Distance to health facility	Distance to a health facility creates barriers in the form of transportation and time costs for users

### Findings

According to the codes indicated in Table 1, the study participants' thoughts on CBHI implementation barriers and strategies to overcome these are described in this section.

### Inadequate population coverage

In Bangladesh, a lack of population coverage hinders the successful implementation of CBHI schemes. Due to the voluntary enrolment approach, a limited number of people were interested in enrolling in these schemes, and even fewer were interested in renewing their membership after their insurance periods had expired. All thirteen participants identified inadequate population coverage as a major impediment to the successful implementation of CBHI. A researcher specified that,

“… *The greatest difficulty is convincing and enrolling a sufficient number of individuals. [Inadequate] enrollment and renewal challenges will arise specifically for small community-based insurance schemes due to their approach of voluntary membership*” (Participant 9)

Interviewees mentioned that membership renewal might be influenced by healthcare benefit consumption and their frequency of healthcare utilization during the membership tenure, satisfaction with the quality of care, and the availability of substitute healthcare choices in their community. According to a majority of participants, members who were unable to acquire enough healthcare benefits from the scheme after purchasing a membership package are unlikely to renew their membership. One participant explained,

“*Several reasons might act behind it [membership renewal]. Maybe they might not have had the opportunity to utilize healthcare.… Moreover, are they happy with the service? or, did they get any alternate option?*” (Participant 1)

To encourage more individuals to join health insurance, participants advised door-to-door visits by community health workers (CHWs), health education sessions, campaigns, and financial assistance to destitute families. In this regard, a monetary incentive for CHWs depending on their performance could incentivize them to visit more households to increase the number of insured members. One respondent mentioned a plan to aggregate funds from the local elite to support struggling families, which may have stimulated their interest in joining the scheme. According to a participant in the CHWs incentive,

“*We set an insurance target [to enroll new members] and if a community health worker can achieve that target, we will reward them, they [community health worker] get motivated and incentivized to achieve the target for the reward*” (Participant 2)

Two interviewees also mentioned that including non-health benefits in the benefits package such as savings opportunities, discounted training programs and grocery items could motivate membership renewal. According to an interviewee,

“…*members were able to buy some grocery items at a discount rate. We listed the regularly needed items and provided them 10% to 30% discount. This step encouraged the members to renew*” (Participant 5)

### High claim rate

High benefit consumption, according to six interviewees, is another potential barrier to the implementation of CBHI in Bangladesh. CBHI schemes, like other health insurance, face moral hazards and adverse selection. Beneficiaries tend to overuse benefits, with some even claiming insurance before they had legitimate needs. Some of the participants stated that since their insurance relies significantly on primary healthcare, particularly maternal health services, those who are pregnant or planning to become pregnant are likely to purchase a membership to receive insurance benefits. One interviewee expressed that,

“*When there is a pregnant woman in a family, if we try to convince them they become interested in the insurance to get the benefits*” (Participant 2)

A similar tendency was also noticed by insurance providers that offer secondary and tertiary level care, who found that consumers are keen to join health insurance before needing healthcare service. In this regard, an interviewee noted that,

“*We observed that only those [ready-made garments] workers were willing to join who needed some complicated or major operation. Because if they give only 300 BDT [as premium], the treatment cost for them will reduce by 50%…*” (Participant 3)

One of the policymakers suggested group health insurance and mandatory enrollment as a way to overcome the obstacles of moral hazard and adverse selection. According to the interviewee-

“*It would be useful if a group-based CBHI strategy could be established and made mandatory by the government to overcome these barriers*” (Participant 11)

### High startup and administrative cost

Seven interviewees discussed high start-up and administrative costs and limited or unavailable financial support from the government or donor agencies as barriers. Despite CBHI schemes being generally managed by NGOs as a not-for-profit service, participants said they could not cover operating costs with insurance revenue. A participant expressed that,

“*We are trying to recover the total cost but we can manage to recover around 80% of it through healthcare [insurance scheme]. Our head office provides the rest of the 20% as subsidies*” (Participant 4)

CBHI insurers must undertake routine advertising and awareness activities, and manual procedures, typically involving a significant amount of work in record keeping, premium collection and depositing in the bank, and claim processing, all of which drive up administrative costs. At the same time, a substantial initial investment necessary for such an insurance program was explained as a barrier to starting an insurance initiative. An interviewee said,

“*We planned to create a cashless system for the service providers and we did it.… but the claim management and claim verifications were tough…. staff from different programs…backed this insurance program but if any organization wants to start this program fresh, the administrative cost will be higher with the hassle.*” **(**Participant 7**)**

To minimize administrative workload and premium collection costs, one participant recommended adopting an automated computerized system and mobile banking payment systems such as bKash, Nagad, and Ucash. During the initial stage, interviewees also emphasized the need for financial assistance from the government or donor agencies as startup capital. One interviewee stated that,

“*At the very first moment, seed money is needed from the donor or the government for 2 years as the premium collection is not enough in the first phase. After running for 2–3 years, then a large pool can be created, and it can sustain in the long run.*” (Participant 5)

Similarly, another respondent emphasized the necessity of government financial and technical assistance. The interviewee further underlined the importance of referral support from government health facilities for the successful implementation of CBHI. According to the interviewee-

“*We need both financial and technical support from the government. We need financial backup as well as referral support from government health facilities*” (Participant 3)

### Scheme design

Members' decisions to join health insurance are also influenced by benefit package design, premium amount, and copayment rates. Four participants discussed scheme features as the implementation barriers in Bangladesh. One participant noted the necessity of including medicine in the benefits package because medicine accounts for the largest share of out-of-pocket expenses in Bangladesh. This may help to increase insurance attractiveness; otherwise, it may be difficult to attract members. Our respondent identified scheme features as a barrier because such insurance typically was targeted and designed to reduce the burden of healthcare expenses on socioeconomically disadvantaged families. According to an interviewee,

“*When rich people get to know that this scheme is targeted for the poor population and the coverage is low, they do not feel interested anymore*” (Participant 7)

Another interviewee cited high copayment rates as a deterrent to enrolling in health insurance. Respondents also stated that, while insurance only provides primary healthcare, members are less likely to purchase their policy because they can seek healthcare from public facilities and get medicine from a pharmacy rather than paying an insurance premium and copayment to consult with a doctor. One researcher described the issue of copayment as,

“*The benefit they get from it [insurance scheme] is not much helpful for them…. patient pays for 80% cost while the insurance scheme bears only 10–20% of the total expense.*” (Participant 8)

The interviewees suggested the use of research-based evidence in constructing an appealing health insurance scheme. They also talked about research on implementation costs to distinguish how much money they would need to collect from their members and how much assistance they would require from the government or donor agencies. Respondents discussed designing a scheme that includes opportunities to consult both public and private healthcare practitioners, which could enhance the likelihood of health insurance. According to a participant,

“*The choices of healthcare provider should be increased. Both the public and private providers should be included in the program*” (Participant 1)

### Competitive healthcare market

Growing informal healthcare professionals, such as village practitioners, traditional healers, and drug sellers, affect health insurance according to the three interviewees. According to one participant, local informal providers do not want their scheme to be popular; therefore, they try to discredit the scheme doctors by spreading rumors about them in the community. An additional hurdle identified by one respondent is that people are accustomed to seeking healthcare from local pharmacies, which prescribe antibiotics to increase profits. This also ensures that patients recover quickly. One interviewee described the issue of informal providers as,

“…*the doctors of our project would not do that [prescribe antibiotics] and, in that case, the recovery might take a few more days. The informal doctors try to grab that chance to create confusion among the village people*.” (Participant 1)

Our participants did not provide any detailed strategies to address the barrier of a competitive healthcare market.

### Inadequate knowledge of health insurance principles and CBHI scheme

All thirteen interviewees identified inadequate knowledge of health insurance principles as a major barrier for CBHI in Bangladesh. They claim that people in their community are not familiar with health insurance concepts, making it more complex to enroll the socio-economically disadvantaged segments as they do not have sufficient health education. A participant described that,

“*The challenge persists from the population perspective in terms of acceptance. The marginalized people think that why do they give money before the service or they think that they are in sound health condition, why do they need to pay for insurance*.” (Participant 3)

Another interviewee discussed this barrier and compared it with micro-credits and savings. According to the interviewee,

“*Insurance as a concept is very unfamiliar in the community. People understand credits and savings. If you start making them understand the health-related uncertainty and the concept of the premium, they would get it for now but when they do not get the premium amount back if they do not get sick, they will not accept the concept because the total practice of insurance is unknown in our community.*” (Participant 6)

Our interviewees proposed making door-to-door visits by engaging the services of community health workers who are already familiar with their community to promote the health insurance scheme. One participant recommended organizing health education sessions by involving influential local personnel so that the community may understand more about health insurance. One of the participants stated,

“*We organize weekly group sessions among different professional groups or in different villages so that they can learn about this scheme and join*” (Participant 5)

In Bangladesh, micro-finance institutions are well known among the lower socio-economic clusters. They know more about microfinance procedures than health insurance. The common practice for this community is to receive loans from the NGOs and then pay back the loan by instalment. One participant quoted the micro-finance initiative taken by BRAC, which goes by the name “Medical Treatment Loan”. Under this initiative, an NGO will provide medical loans instead of asking for advance premiums for health insurance. According to the participant,

“…*a borrower of BRAC microfinance scheme or anyone from his family gets sick, then… with BRAC slip they will get a 50% discount for consultation, and the estimated cost of treatment will be written on the other side of that slip. Then the patient will go back to the BRAC microfinance office with the slip and it will be appraised for how much loan he/she might get. We saw a huge response in the first year of piloting and the recovery rate was 98.5%* ….” (Participant 6)

### Quality of care and trust

Seven participants talked about how the quality of healthcare services and trust in insurance providers are two factors that make health insurance more difficult to implement. The availability of healthcare providers or drugs could also impact health insurance implementation in the long run. According to one of the interviewees,

“…*If the doctor remains absent when she [insured member] visits the hospital, then that family will lose their interest in our insurance. If we can ensure the quality of our service and increase the efficiency of our staff, then insurance [member enrolment and renewal] becomes very easier.*” (Participant 2)

Similarly, if pharmacies or enrolled drug stores remain closed during patients' visits, member satisfaction and trust in insurance providers will be affected. Participants also noted infrastructural and workforce constraints on both the health and management areas as hurdles to their scheme. Health workforce shortage was brought up by one of the participants,

“…*under each regional office, there are 10–12 health centres. At least 2 MBBS doctors should be there in each region but there are less than 12 MBBS doctors in 12 health centres*” (Participant 4)

Participants also talked about the barriers they face regarding healthcare providers' behaviour. They claim that providing poor quality services and misbehaviour by clinicians with patients from insured families will reduce community acceptance and may lead to patients not continuing with the scheme. Furthermore, informal providers have been working in rural areas for a long time and are well-liked in their community; trust is most important to health insurance when replacing informal providers’ roles in the community. According to one of the participants,

“*In the rural areas, people often seek healthcare from village doctors, quacks, pharmacies etc. In that case, when we want to divert them, then there is a trust issue*” (Participant 1)

Our interviewees proposed several ways for overcoming barriers linked to care quality and trust. Two of our participants advocated for the use of more paramedics to enhance the supply of healthcare professionals, or even paramedics who could relieve physicians from some of their burdens so that they can see more patients that are most in need. They also recommended telehealth services and online consultations to increase accessibility and build trust within the community. An interviewee explained that,

“*When the doctor is absent… but the patient demands the doctor's consultation, the paramedics establish the connection with the doctor through skype. If anyone wants to utilize this option, he/she can contact the doctor even after hours and in that case, he can directly call the doctor through skype. In this way, we are trying to build trust*.” (Participant 1)

They also talked about the importance of securing the supply of high-quality drugs and providing incentives for healthcare providers to ensure that members receive high-quality care. This problem was explained by one participant as follows,

“*Controlling this issue seemed quite difficult to us because the agenda of the medical practitioner is different…might not think himself accountable to consult with the cardholder patients on priority basis as he does not have any additional benefit for this. It is necessary to design a proper mechanism for this issue such as to incentivize the medical practitioners*” (Participant 6)

### Distance to a health facility

Three of our interviewees noted distance to a health facility as an implementation barrier for health insurance because it is connected with additional travel and time costs from the user’s perspective. They stated that the distance between a health facility and a member's house reduces the likelihood of being insured. As one participant described it,

“…*a village a bit far away from here [health facility] …One will need 150–200/- [Bangladeshi taka] to come here from that place! But if he [she] wants to visit the government hospital, it will cost him [her] only 10/- [Bangladeshi taka] …*” (Participant 2)

One of our interviewees proposed implementing satellite clinics because this approach helped to get the elderly population insured in their scheme. As stated by the participant,

“*We started a satellite clinic in the rural areas. Every week the satellite clinic covered different village areas……[they] liked this service very much and the elderly group was also coming to the satellite clinic*” (Participant 5)

### Views of policymakers on CBHI

Our interviewees from the representative of CBHI schemes explained the need for technical and financial support from the government. In this section, we will discuss the policymakers’ views and plan for the CBHI schemes.

Four of our policymakers acknowledged that the government is eager to provide technical and policy-related assistance to CBHI initiatives. In addition, they mentioned that the government is currently providing technical assistance to several privately initiated health insurance. According to a policymaker-

“*We are indirectly involved with a few of them, including an insurance scheme for ready-made garment workers and another for tea-garden workers. The Ministry of Health and the Health Economics Unit are collaborating to provide technical support to those who want it*” (Participant 10)

The policymakers also specified the ongoing government plan for CBHI schemes. They discussed the significance of the small CBHI schemes that are currently functioning in Bangladesh and the necessity to centralize their administration. A policymaker told as-

“*Most country's [insurance] initiatives were taken on a small scale before being pooled together under a single platform. Since integrating everyone under one umbrella takes time, small initiatives should be undertaken in the meantime*” (Participant 12)

All policymakers expressed the necessity for a separate government entity to facilitate healthcare financing initiatives, including CBHI schemes. Therefore, they discussed continuing activities for the formation of the National Health Security Act and the National Health Security Office. According to an interviewee-

*“The act [National Health Security Act] is now being designed and already incorporates CBHI. The concept of cooperatives [CBHI] has existed here for a very long time. Until it is governed by a central authority, it will not be effective*” (Participant 13)

## Discussion

Our research examines the barriers to implementing CBHI in Bangladesh and stakeholders' views on strategies to overcome these barriers. One of the key issues of CBHI schemes discussed by our participants is insufficient population coverage. Low enrolment and renewal rates were also noted as barriers to the successful implementation of CBHI, which they believe is due to the voluntary nature of membership. CBHIs are often small in size because they are designed to target a specific geographic area. Such schemes are more prone to implementation issues since they have limited risk diversification opportunities due to small membership pools. Previous studies indicate that awareness and understanding of the CBHI concept, trust in scheme management, perceived quality of care, and socio-demographic characteristics all play a role in households' decision to join and stay in the scheme [20, 21, 30, 31].

To encourage individuals to join CBHI, our interviewees suggested door-to-door visits, health education sessions, campaigns, and financial assistance to the destitute. Previous studies have discovered that door-to-door visits, health education sessions, and campaigns have an effective influence on increasing enrolment and renewal [33–41]. Similarly, providing some form of financial assistance to disadvantaged households could make it more affordable for them to join CBHI [29, 33, 34, 36, 42, 43]. Our stakeholders also talked about providing additional performance-based monetary incentives to CHWs to motivate them to visit more households and recruit new members. Previous research in Bangladesh has shown that monetary incentives positively impact CHW’s retention and intervention success [44, 45]. Our interviewees also suggested incorporating non-health benefits in the benefits package to encourage their membership renewal. In Bangladesh, several schemes have attempted to implement non-health benefits in CBHI among informal workers [18] and ready-made garment workers [46]. However, the impact of non-health benefits is yet undetermined in Bangladesh. According to a study in Vietnam, the inclusion of non-health benefits did attract more individuals and stimulated the expansion of the social health insurance scheme [47]. Similarly, another research suggested that countries must push beyond the health sector to achieve UHC, and social health insurance schemes may need to incorporate non-health benefits to get maximum insurance coverage [48]. The inclusion of non-health benefits may serve as an incentive for Bangladeshi informal workers to join and renew CBHI memberships.

Moral hazard is a recognized threat that leads to high benefit consumption and obstructs voluntary CBHI implementation in Bangladesh. Due to the voluntary nature of the schemes, they are sensitive to adverse selection, in which high-risk and sick people are more likely to purchase health insurance than low-risk and healthy people [49]. Adverse selection is a threat to financial viability because it limits the potential for risk-sharing from healthy to sick people, forcing insurers to raise premiums to adjust costs when they face high claims. Low-risk persons are more likely to leave a scheme as a result of the increased premiums due to the expected low healthcare costs relative to their CBHI contribution, resulting in further adverse selection [50]. Adverse selection has been documented in CBHI schemes in India [51], China [52], and Africa [49]. Participants also emphasized the moral hazard problem, which states that once people are insured, they are more prone to overuse medical services. This might be due to either a lack of concern for one's health after joining a scheme or deliberate deceitfulness by consumers. Introducing group coverage, mandatory membership, and waiting periods are recognized techniques to overcome the problem of adverse selection; however, claim limits and copayments could lessen moral hazard [53]. Further research is suggested to explore the effectiveness of those strategies on moral hazard and adverse selection.

Our interviewees noted that the progress of CBHI schemes is significantly impacted due to high startup and administrative costs. The establishment of CBHI is costly as it necessitates new infrastructure, staff recruitment, and the development of procedures and regulations before receiving significant premiums, making it difficult to recoup expenses. Studies conducted in Bangladesh stated that none of the CBHI with voluntary membership could recover their operating costs and heavily rely on subsidies [13, 15]. Stakeholders stated that CBHI requires regular marketing and awareness campaigns and efforts to collect premiums, creating additional administrative and financial barriers. The most costly administrative task of CBHI in Tanzania was revenue collection comprising marketing and registration activities [54]. Interviewees also acknowledged how they are challenged by the absence of additional monetary assistance from the government or donor agencies. Since premium and co-payment arrangements are insufficient, the financial viability of such schemes is a major concern.

Furthermore, due to the COVID-19 crisis, external funding for CBHI appears to be becoming increasingly difficult in the future. Interviewees advised adopting an automated computerized system and a mobile banking payment system to reduce workload and administrative costs. In the health insurance market, new technology and innovation, such as automation [55] and mobile money payment [56] are very effective in decreasing manual workload, time, and transition costs, but we see no evidence that this can entirely obviate the need for external financial support. Our participants also highlighted the need for a risk fund allocation for CBHI and the need for seed money to get a CBHI off the ground.

Our study found that scheme features, i.e., benefits package, could be a challenging factor to attract the target population. Due to a limited risk pool and inadequate population coverage, most benefit packages are not adequately designed to cover all medical expenses. As a result, insured members frequently have to spend a large amount of out-of-pocket money for medical diagnostics and medication as a co-payment. Our interviewees discussed the importance of including medicine in the benefits package and designing the benefit package to meet people's healthcare needs and preferences. The scheme should be affordable to the poor while also appealing to the wealthy, cover both inpatient and outpatient services, and provide a choice of public and private providers. The benefits packages which can address the needs of a community, are equitable, and provide outpatient services are likely to increase enrollment [57–59]. On the other hand, low membership is attributed to chronic disease exclusion [60], a high premium amount [60], and limited disease coverage [57]. Our participants advised that benefit packages be designed using research-based knowledge to attract more customers.

While people do not understand the concepts of medical insurance, they are more likely to decline to join or continue a CBHI policy. According to our study, the community's awareness of health insurance, socio-cultural beliefs, social norms, and healthcare-seeking patterns could impede CBHI implementation. The concept of health insurance is unfamiliar in Bangladesh, particularly among the disadvantaged rural population. Low enrolment in CBHI appears to be a strong sign that the targeted rural populace does not realize the value of an advance payment, i.e., purchasing a specific measure of assurance against an unpredictable future event. A review article on CBHI articulated that proper understanding of the concept and principles of health insurance has a positive impact on enrolment and renewal in different countries such as Afghanistan, Cameroon, China, Ghana, Guatemala, India, Kenya, Nigeria, the Philippines, Tanzania, and Uganda [31]. Interviewees suggested a promotional campaign, awareness-building activities such as door-to-door visits, and health education sessions in the community with the involvement of local community health workers and influential personnel to promote the insurance scheme by educating people.

Moreover, medical treatment loans mentioned by our interviewee could be a feasible variation of community health insurance in Bangladesh. Microfinance scheme borrowers can apply for medical loans for themselves and their family members who have medical needs. The microfinance institute will allow them to pay the debts in instalments. Further research is needed to explore the community’s response, acceptability, and the prospect of a medical loan-based micro health insurance scheme.

Trust in insurance providers and consumer satisfaction, influenced by perceived healthcare quality, were mentioned by respondents as barriers to CBHI implementation. The availability and efficiency of healthcare personnel, their attitude or engagement with patients, and health facility features all play a role in how people perceive healthcare quality [30]. Low-quality healthcare because of insufficient or unavailability of healthcare professionals and drug supplies during patients' visiting hours, infrastructural features, and health professionals' negative attitudes were identified as significant barriers to CBHI implementation in our study. Low-quality healthcare and healthcare providers’ inefficient technical proficiency were barriers to enrolment and membership renewal in Rwanda [61]. Another study in Benin reported that approximately 30% of members drop out of a scheme due to the providers’ negative attitudes and behaviours [37]. Most of the stated reasons for dissatisfaction were long waiting queues, providers’ negative attitude and efficiency, reimbursement rates, membership fees, drug quality, and treatment variation among socio-economic classes [30]. The capacity to provide high-quality healthcare services based on the needs of enrollees would boost their satisfaction and build trust over time. Our interviewees suggested paramedics, telehealth services, and online consultation to improve the healthcare supply. Incentives and training are proposed to motivate health professionals and the provision of high-quality medicines to ensure consumer satisfaction and trust.

The distance between a household and a health facility is an important indicator of inequality in terms of healthcare accessibility. A long distance has been cited as a barrier to implementing CBHI in Bangladesh, potentially discouraging the target population from participating. According to studies, low enrolment has been linked to high travel costs due to great distances [37, 61]. A study in Bangladesh also found that households close to health facilities were 2.7 times more likely to renew their membership [21], which could be due to the information gap and travel expenditure to access healthcare services. To solve the issue of long distances, one of our participants suggested setting up a satellite clinic; however, satellite clinics are only effective for outpatient healthcare services. Collaboration between public and private providers could open up possibilities to receive healthcare from nearby facilities, making healthcare more accessible. Although our study gathers experience from Bangladesh, our findings, broader discussion, and suggested strategies apply to other low-resource contexts and are useful to policymakers.

### Limitations

Although the study was qualitative, it may have benefited from quantitative insights as well as scheme beneficiaries’ participation. However, it was challenging to explore the beneficiaries’ perceptions of CBHI because of COVID-19 restrictions. Secondly, our study only included health insurance schemes whose information was available in the literature, which could be a source of bias given that successful schemes are more likely to be documented [14]. Furthermore, we may have lost some information because some of the interviews were disrupted due to a poor internet connection. Despite these limitations, we believe this study provides a comprehensive understanding of the implementation barriers of CBHI in Bangladesh.

## Conclusion

This qualitative study examines the barriers to implementing CBHI in Bangladesh from the viewpoints of insurance providers, researchers, and policymakers, including respondents from several active and recently closed schemes, as well as researchers and policymakers. The key barriers to CBHI implementation are insufficient population coverage, adverse selection, moral hazard, high startup and administrative costs, lack of knowledge of health insurance modalities, inadequate healthcare supplies, insurer trust, and the distance to travel to health facilities.

The CBHI in Bangladesh could serve as a foundation for the planned National Health Security Act towards UHC. Bangladesh is the birthplace of microfinance, and we have seen several integrations between health insurance and microfinance schemes. However, public–private partnership initiatives are necessary to reach a significant portion of the target population. Microfinance institutes and cooperative societies working at the grassroots level in Bangladesh can be prospective partners because they already have a well-organized and trusted platform. Our findings strongly suggest that policymakers should support CBHI schemes in technical and financial aspects and establish a distinct body to investigate the efficacy of public–private (i.e., NGO, microfinance, and cooperative societies) partnered approaches to CBHI in Bangladesh.

## Supplementary Information

**Additional file 1.**

## Data Availability

The topic guide used in this study is provided as an additional file 1, further inquiries on data/materials analyzed in this study are available upon request to the corresponding author. The framework matrix with anonymise filled information is also available upon request to the corresponding author.
