# Supplementary material for: Implementation barriers and remedial strategies for community-based health insurance in Bangladesh: insights from national stakeholders
Source: BMC Health Serv Res. 2022 Sep 24;22:1200. doi: 10.1186/s12913-022-08561-7 (PMC9508716; doi:10.1186/s12913-022-08561-7)
Supplement: Supplementary file 1 — Additional file 1. [file 12913_2022_8561_MOESM1_ESM.docx]

**Name of department: Department of Management Science, School of Business.**

**Title of the study:** Challenges and prospects of Community-Based Health Insurance Schemes in Bangladesh.

## Please tick the box if you agree with the statement

- I confirm that I have read and understood the Participant Information Sheet for the above project and the researcher has answered any queries to my satisfaction.
- I confirm that I have read and understood the Privacy Notice for Participants in Research Projects and understand how my personal information will be used and what will happen to it (i.e. how it will be stored and for how long).
- I understand that my participation is voluntary and that I am free to withdraw from the project at any time, up to the point of completion, without having to give a reason and without any consequences.
- I understand that I can request the withdrawal from the study of some personal information and that whenever possible researchers will comply with my request. This includes the following personal data:
  - audio recordings of interviews that identify me;
  - my personal information from transcripts.
- I understand that anonymized data (i.e. data that do not identify me personally) cannot be withdrawn once they have been included in the study.
- I understand that any information recorded in the research will remain confidential and no information that identifies me will be made publicly available.
- I consent to being a participant in the project.
- I consent to being audio recorded as part of the project.

| (PRINT NAME) | Hereby agree to take part in the above project |
| --- | --- |
| Signature of Participant: | Date: |

1. Does the health insurance scheme (schemes) face any operational challenges? If yes, what are those challenges?

***Probes:***

*Are there any administrative challenges faced by this your scheme?*

*Does the enrolment and renewal rate sufficient to sustain?*

*Do you face any demand side barriers such as lack of awareness, knowledge and trust on health insurance? Is there any distance related issue raised by the consumers?*

*Do you have enough human resources and infrastructure?*

*Does this scheme have any referral system or network with other hospitals?*

1. How can those challenges be overcome?
2. Do you think this scheme (schemes) is (are) financially feasible and possible to scale up?

***Probes:***

*If not, how to make it (those) financially feasible?*

*Is there any need for internal support (either financial or technical) to make it viable?*

1. Can you describe ongoing and planned government health care financing reform activities?
2. How the CBHI to be integrated with those ongoing or planned healthcare financing reform activities?
3. Please explain what could be the key obstacles to implementing CBHI in large scale in Bangladesh?
4. Can you explain from your experience, how can those obstacles be overcome?
